# Application of Nanozymes in Environmental Monitoring, Management, and Protection

**DOI:** 10.3390/bios13030314

**Published:** 2023-02-24

**Authors:** Miaomiao Wang, Ping Zhu, Shuge Liu, Yating Chen, Dongxin Liang, Yage Liu, Wei Chen, Liping Du, Chunsheng Wu

**Affiliations:** 1 Institute of Medical Engineering, Department of Biophysics, School of Basic Medical Sciences, Health Science Center, Xi’an Jiaotong University, Xi’an 710061, China; 2 Key Laboratory of Environment and Genes Related to Diseases, Xi’an Jiaotong University, Ministry of Education of China, Xi’an 710061, China

**Keywords:** nanozyme, sensing, monitoring, environmental pollutant, catalytic activity

## Abstract

Nanozymes are nanomaterials with enzyme-like activity, possessing the unique properties of nanomaterials and natural enzyme-like catalytic functions. Nanozymes are catalytically active, stable, tunable, recyclable, and versatile. Therefore, increasing attention has been paid in the fields of environmental science and life sciences. In this review, we focused on the most recent applications of nanozymes for environmental monitoring, environmental management, and environmental protection. We firstly introduce the tuning catalytic activity of nanozymes according to some crucial factors such as size and shape, composition and doping, and surface coating. Then, the application of nanozymes in environmental fields are introduced in detail. Nanozymes can not only be used to detect inorganic ions, molecules, organics, and foodborne pathogenic bacteria but are also involved in the degradation of phenolic compounds, dyes, and antibiotics. The capability of nanozymes was also reported for assisting air purification, constructing biofuel cells, and application in marine antibacterial fouling removal. Finally, the current challenges and future trends of nanozymes toward environmental fields are proposed and discussed.

## 1. Introduction

Most life activities in nature involve enzymes. Natural enzymes are macromolecular biocatalysts composed of most proteins and a few nucleic acids that run through the metabolism of life [1]. They have high catalytic efficiency, good substrate specificity, and biocompatibility [2]. Therefore, they are widely used in various fields, including disease diagnosis, clinical treatment, agricultural engineering, paper and leather, textile industry, and food processing. However, most natural enzymes are easy to inactivate or their activities are inhibited under nonphysiological conditions, which severely limits the wide application of enzymes. In addition, enzymes also have defects such as storage stability and recovery difficulties, complex production as well as purification processes, and high costs [3,4]. With the rapid development of nanoscience and life science, simulating the structure and catalytic activity of natural enzymes to construct substitute products has gradually become a new direction in which to expand the application of natural enzymes.

The term “artificial enzymes” was coined by Ronald Breslow for enzyme mimics. An “artificial enzyme” combines a metal catalytic group and a hydrophobic binding cavity [5]. In 2004, Scrimin and his colleagues created the term “nanozyme” and used gold nanoparticles functionalized by triazetidine as the catalyst for the transphosphorylation reaction [6]. In 2007, Yan’s team found that magnetic nanoparticles (Fe_3_O_4_ MNPs) have a catalytic activity similar to horseradish peroxidase (HRP), indicating that some inorganic nanoparticles can also have peroxidase-like properties [7]. In 2013, Wei and Wang used the term “nanozyme” to describe some nanoscale materials with enzyme-like characteristics, namely, nanozyme is a kind of nanomaterial with similar natural enzyme catalytic activity and enzymatic reaction kinetics [8]. In the ten years since then, nanozyme experienced a period of rapid development and application in various fields. Based on advanced nanotechnology, a variety of nanozymes with catalytic activity comparable to that of natural enzymes have been explored. Compared with natural enzyme, nanozyme has the following obvious advantages: (I) High stability: inorganic nanomaterials are less fragile than natural enzymes, which enables the use of nanozymes under a wide range of pH (3–12) and temperature (4–90 °C) conditions. In contrast, natural enzymes are usually inactivated under extreme pH and temperature conditions. (II) Low cost: the production process of enzymes is usually complex and expensive, while inorganic nanomaterials are easy to produce, with high efficiency and low cost. (III) Recycling: Nanozymes are recyclable, and there is no substantial loss of catalytic activity in subsequent cycles. (IV) Easily multifunctional: Nanozymes have sufficient surface area to allow them to be coupled with multiple ligands to achieve multifunctionability [9]. (V) High catalytic activity: The level of activity is comparable to that of biological enzymes with the help of advanced nanotechnology. At the same time, a variety of factors influence the level of activity such as size, shape, composition, crystal surface, charge, and hydrophilicity. Although nanozymes have the above advantages, some nanozymes have the disadvantages of toxicity, low specificity, and poor dispersion. During the degradation and protection of environmental pollutants by nanozymes, nanozymes inevitably contact with water, animals and plants, soil, and air. Therefore, the safety of nanozymes is crucial. For example, some heavy metal nanozymes (Au, Cu, Ce, Fe, etc.) will be absorbed into soil and water, causing ecological pollution [10]. The continuous enrichment of heavy metals will eventually endanger human health through the food chain. In addition, graphene, quantum dots, copper–carbon dots and other nanomaterials have their own toxicity, and their dispersion will be strengthened during trial, which will make them more easily absorbed by aquatic plants and water bodies [11]. Toxicity can be reduced by reducing the size of nanozyme [12]. In addition, we can control the surface charge of nanozyme to regulate its permeability to cells in the human body [13]. The metal core is the source of toxicity, so it can be prevented from leakage by mixing other metal ions and chemical sealing [14,15].

**Figure 1:**
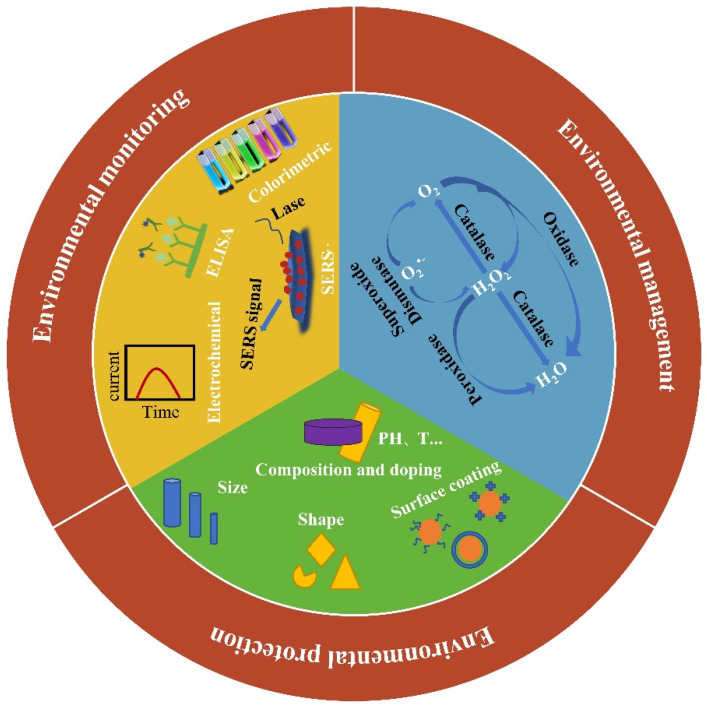
Nanozyme as a promising tool from environmental monitoring and environmental management to environmental protection.

The International Enzyme Commission (I.E.C.) specifies that natural enzymes can be classified into six categories by the enzymatic reaction as oxidoreductases, hydrolases, isomerases, lyases, ligases, transferases. Since nanozymes are a class of nanomaterials that mimic the catalytic activity and enzyme kinetic characteristics of natural enzymes, the categories also similar to that of natural enzymes could divide nanozymes into the following six categories: redox nanozyme, hydrolyzing nanozyme, lytic nanozyme, transfer nanozyme, isomeric nanozyme, and linked nanozyme. Currently, the reported nanozymes are mainly in the redox nanozyme family, whose members are oxidase (OXD) [16], peroxidase (POD) [17], catalase (CAT) [18], and superoxide dismutase (SOD) [19] (Figure 1). Oxidase catalyzes the oxidation of substrates using oxygen as the electron acceptor. Subsequently, O_2_ is reduced to water or hydrogen peroxide. Peroxidase nanozyme can catalyze peroxides whose substrate is usually used as an electron donor. In biomedicine, it defends against pathogens and removes the toxicity of reactive oxygen [20]. Catalase often catalyzes H_2_O_2_ to produce oxygen and water. It is found that many metal materials and even metal oxides have catalase-like activity. Superoxide dismutase disproportionates superoxide radicals into oxygen and hydrogen peroxide and alleviates oxidative stress generated from cell metabolism. There is also a family of hydrolyzing nanozymes including phosphatases [21], nucleases, and proteases. They catalyze the separation of phosphate groups and the hydrolysis of phosphate diester bonds and peptide bonds. The family of lytic nanozyme comprises the carbonic anhydrase [19]. Nanozymes mostly catalyze the optical signal transmission of chromogenic substrates, so that other three types of nanozymes are rarely reported. It is hoped that the design of nanozymes can break through more types and functions limitations.

The reported nanozymes can be classified into four categories according to the materials, such as metal-based, metal oxide-based, carbon-based, MOF-based, and other material-based. Currently reported carbon-based nanozymes include fullerenes, carbon nanotubes (CNTs), graphene, graphene oxide (GO), carbon dots (CDS), graphitic quantum dots (GQDS), and carbon nitrides [22]. Because of their special electronic and geometric properties, they can mimic the catalytic center of natural enzymes and possess catalytic activities such as oxidase, peroxygenase, superoxide dismutase, and catalase. On account of the intrinsic enzymatic activity of carbon nanomaterials, they can be combined with other materials or modified and functionalized to enhance enzymatic activity. Ye et al. [23] reported a highly specific N-doped nanozyme with HBF as a porous carbonaceous and nitrogen-containing precursor to prepare N-doped carbon nanozymes named HBF-1-c800 by high temperature pyrolysis with N-doping efficiency up to 5.48%, which is higher than the value of most reported N-doped carbon nanozymes. As a result, they found that the apparent POD activity of HBF-1-c800 shows a three to seven-fold enhancement over traditional carbon nanozymes and a five-fold enhancement over the reported N-doped graphene. Consequently, it has been widely used in environmental monitoring and environmental remediation. Although slightly inferior to metal-based nanozymes in terms of catalytic activity, its catalytic activity has been improved, and some excellent designed carbon-based nanozymes show comparable or even better results than that of natural enzymes. 

Metal-based nanozymes are one of the most widely used nanozymes. They have unique optical and electrical properties at the nanoscale as well as excellent catalytic properties [24] and exhibit good activity tunability and high stability. They have been found to exhibit a variety of enzyme-like properties, including oxidase-, peroxidase-, catalase-, and/or superoxide dismutase-like activities [25]. Metallic nanomaterials commonly include Au, Ag, Pt, PD, Rh, Ru, and Ir. For metal-based nanozymes, the catalytic mechanism arises from the adsorption, activation, and electron transfer of the substrate onto the metal surface, in contrast to the mechanisms occurring by changes in the metal valence of the nanomaterial, as in the case of other metal compound-based nanozymes [22]. Studies have found that the surface state of metal-based nanomaterials is one of the important factors affecting their catalytic activity. Therefore, much attention has been focused on how to optimally control the surface of metal-based nanozymes for high electrical conductivity. It can be classified into monometallic nanozymes, bimetallic nanozymes, and multimetallic nanozymes, which are distinguished, as the name implies, by having several metal cores. Bimetallic nanomaterials (BNMS) usually exhibit stronger catalytic performance than monometallic nanomaterials due to the synergistic effect [26], among which platinum-based BNMS have been extensively studied in the field of catalysis for many years. Multimetallic NPs composed of at least three different metals have more possibilities in modulating the activity, selectivity, and stability of surface catalyzed reactions. Hence, the rational design and controllable synthesis of multimetal nanozymes are of great significance. 

Transition metals other than noble metals (Ti, V, Cr, Mn, Fe, Co, Ni, Zn, Al, Mo, and W) usually exist stably in the form of their complex ions, among them the oxide form, thus constituting metal oxide-based nanozymes. Metal oxide-based nanozymes including CeO_2_, Fe_2_O_3_, Fe_3_O_4_, Co_3_O_4_, Mn_2_O_3_, and Mn_3_O_4_ all exhibit multienzyme activities, such as peroxidases, oxidases, hydrolases, and catalases. In addition, they show other physicochemical properties such as fluorescence quenching, dielectric properties, and magnetism [27]. It is worth noting that most metal oxide nanomaterials exhibit lower Km than HRP, which provides the possibility for a wide range of applications. Metal-organic frameworks (MOFs) are porous coordination crystalline materials formed by the self-assembly of metal ions (or metal clusters) and organic ligands through the principles of coordination chemistry [28]. In recent years, some MOFs exhibit their own good enzyme mimicking properties, mimicking the functions of a variety of enzymes, including oxidases, peroxidases, catalases, superoxide dismutases, and hydrolases. The high specific surface area, homogeneously dispersed active sites, structural diversity, and pore size tunability of MOFs can facilitate the efficient contact of reaction substrates to the catalytic sites and, in turn, enhance the catalytic efficiency of subsequent processes. Therefore, its pore size, size, modification, and composition are important factors for regulating enzyme activity. In addition, it can also participate in regulation by external conditions pH, temperature, H_2_O_2_, and so on [29]. The development of other nanomaterials with different structures and properties has provided new sources for artificial enzyme research, such as perovskites, metal sulfides, metal, dichalcogenides, methyl hydroxides, metal phosphates, and polymeric nanostructures [22]. The development of other types of nanomaterials has provided new sources for artificial enzyme research, such as perovskites, metal sulfides, metal, dichalcogenides, methyl hydroxides, metal phosphates, polymeric nanostructures, and others. They can also mimic the enzymatic activity properties of the oxidoreductase family and have received much attention for their unique structures or properties that are different from those of carbon-based nanomaterials, metal-based nanomaterials, and metal oxide nanomaterials and applications in environmental monitoring and remediation. As a carbon nitride, MXENEs have large surface areas, metallic conductivity, antimicrobial activity, and biocompatibility [30] and have been found to possess intrinsic peroxidase-like and oxidase-like activities and can be enhanced by single stranded DNA (ssDNA) adsorbed onto nanosheets. Li et al. constructed a simple label-free colorimetric sensing platform for TB-selective detection based on Ti_3_C_2_@ssDNA. The sensor exhibited good selectivity and sensitivity with a wide linear range of 1.0 × 10 ^−11^ to 1.0 × 10^−8^ m and a low detection limit of 1.0 × 10^−11^ M [31].

In this review, we summarize the most recent applications of nanozymes for environmental monitoring, environmental management, and environmental protection (Figure 1). We firstly introduce the tuning catalytic activity of nanozymes according to some crucial factors such as size and shape, composition and doping, and surface coating. Then, the application of nanozymes in environmental fields is introduced in detail. Nanozymes can not only be used to detect inorganic ions, molecules, organics and foodborne pathogenic bacteria but are also involved in the degradation of phenolic compounds, dyes, and antibiotics. The capability of nanozymes was also reported for assisting air purification, constructing biofuel cells, and application in marine antibacterial fouling removal. Finally, the current challenges and future trends of nanozymes toward environmental fields is proposed and discussed.

## 2. Tuning Catalytic Activity 

Nanozymes are alternatives to natural enzymes but remain slightly inferior in catalytic activity. Thus, we need to focus on several important factors that affect the enzymatic activity of nanozymes as well as current strategies to enhance activity, thereby laying a theoretical foundation for the design of nanozymes.

One of the distinct features of enzymes are their ultrahigh reaction rate. Correspondingly, nanozymes with comparable or even superior activity are long-standing pursuits. Two strategies are discussed here to improve the activity of nanozymes: (I) increasing the inherent activity by delicate design and (II) boosting the activity by confinement effect or external stimulators [32]. The main factors affecting the intrinsic activity of nanozyme are size, composition, doping, shape, and surface modification. External stimulus factors, such as pH, substrate concentration, temperature, and light, affect the catalytic activity. Factors determining the activity of nanozymes need to be optimized for specific conditions in order to achieve maximum efficiency in applications involving detection of target analytes.

### 2.1. Size and Shape

**Figure 2:**
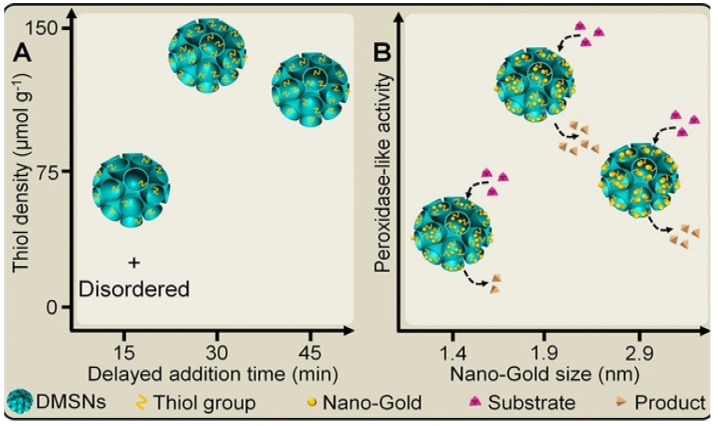
Schematic illustration of the effects of (**A**) delayed addition time on the structures of the final product and (**B**) Nano-Au size on the peroxidase-like activity [35]. Reproduced with permission from Ref. [35], Copyright 2019 American Chemical Society.

Size, shape, and atomic arrangement can lead to changes in the catalytic performance of materials. It was found that the catalytic activity and stability of nanozyme increased with the increase in surface volume ratio. For example, Valden et al. [33] prepared gold clusters with a diameter of 1 to 6 nm on the single crystal surface of titanium dioxide under ultrahigh vacuum to investigate the size dependence of their low-temperature catalytic oxidation of carbon monoxide. It was found that the gold cluster with the largest carbon monoxide oxidation activity was 3 nm. In another case, Zhou et al. [34] used Au nanoparticles with various sizes (2–15 nm) to catalyze the reduction of resazurin, showing that Au nanoparticles of 6 nm exhibited the highest activity. However, small gold nanoparticles tend to aggregate and lose their activity. Scientists often anchor gold nanoparticles to carbon, silica, graphene, and other supporting materials to improve the dispersion of bare Au. Kalantari et al. [35] adjusted the delayed addition time of the thiolated organosilica precursor to control the nanostructure and the thiol density. Moreover, for the first time, they demonstrated that the peroxidase-like activity of T-Dendritic Mesoporous Silica Microspheres (DMSNs)-Au depended on nano-Au size. In addition, the highest activity was achieved at the Au particle size of 1.9 nm (Figure 2).

The catalytic performance of nanozymes can also be modulated by adjusting the shape of the nanostructures. Biswas et al. [36] compared the catalytic efficiency of gold nanorods (GNRs), gold nanoparticles (GNPs), and horseradish peroxidase (HRP). It was proved that the peroxidase activity of gold nanorods with a length diameter ratio of 2.8 was 2.5 times higher than that of HRP and gold nanoparticles, which showed stability in a wide range of pH and temperature. Based on this, a colorimetric sensor for malathion was developed, whose sensitivity of the assay was 1.78 μg/mL. A comparative study of VO_2_ nanoparticles with different morphologies (nanofibers, nanosheets, and nanorods) was conducted and applied to the sensitive colorimetric detection of H_2_O_2_ and glucose by Tian et al. [37]. According to the typical Michaelis–Menten curve obtained for VO_2_ nanozymes, the apparent K_M_ values of VO_2_ nanofibers with H_2_O_2_ as the substrate were lower than that of VO_2_ nanorods and VO_2_ nanosheets. It shows that the VO_2_ nanofibers have a higher affinity for H_2_O_2_ compared with VO_2_ nanosheets and VO_2_ nanorods. Moreover, compared with VO_2_ nanorods and VO_2_ nanosheets, the VO_2_ nanofibers demonstrated the most sensitive response during the H_2_O_2_ and glucose sensing.

### 2.2. Composition and Doping

Some researchers have shown, based on the synergistic effect, that combining a variety of nanomaterials or conjugating several nanomaterials to form a hybrid can provide a catalytic center [38], improve the electron transfer between the nanozyme and the substrate, and generate additional active sites, which can adjust the catalytic activity of the catalyst.

Zhu et al. [39] combined TiO_2_, CuInS_2_, and CuS into a ternary metal sulfide-based hybrid. Owing to the synergistic effect among TiO_2_, CuInS_2_, and CuS components, compared with the control sample of Fe_3_O_4_/rGO, TiO_2_/rGO, Fe_3_O_4_, TiO_2_, and rGO, the prepared TiO_2_/CuInS_2_/CuS nanofibers showed excellent peroxidase (POD)-like activity. They subsequently developed a sensor for the detection of dopamine with a detection limit of 1.2 μM. Wang et al. [40] incorporated iron oxide nanoparticles (Fe_3_O_4_NPs) into the heterodimer composed of gold and platinum to form a hybrid nanomaterial with good peroxidase-like activity. The formation of an alloy between platinum and gold can significantly improve the activity and selectivity of platinum-based catalysts. The nature of the peroxidase-like activity of the Fe_3_O_4_@Au-Pt hybrid nanomaterial originates from their ability to transfer electrons between the reducing substances and H_2_O_2_. The colorimetric sensor with a lower detection limit of 0.0018 μM was developed for glucose. Another form of composition is loading. Zhao et al. [41] covalently fixed the carbon point (C-dots) on the inner surface of the amino terminated dendritic silica sphere (dSs) while coupling the gold nanoclusters (Au NCs) on the outer surface. It not only maintains the superoxide dismutase-like enzyme activity of the carbon point but also improves the peroxidase-like-activity of the gold nanoparticles. Furthermore, adjusting the loading ratio of the two kinds of nanozymes can meet different functional requirements.

### 2.3. Surface Coating

The surface modification of nanozyme not only plays a connecting role in the combination of nanomaterials but also is of great importance to the regulation of catalytic activity. The surface catalytic reaction process can be described by several basic reaction steps, including substrate adsorption, substrate diffusion on the surface, chemical reaction, and then product desorption to regenerate the active site [42]. Each step will be affected by surface modification. Thus, some general strategies can be adopted for surface modification, such as changing the electronic structure of the surface, regulating the surface acidity, blocking surface contact, promoting product desorption, mediating the exposure of active sites to regulate substrate binding, and applying effective methods for surface electronic structure. 

**Figure 3:**
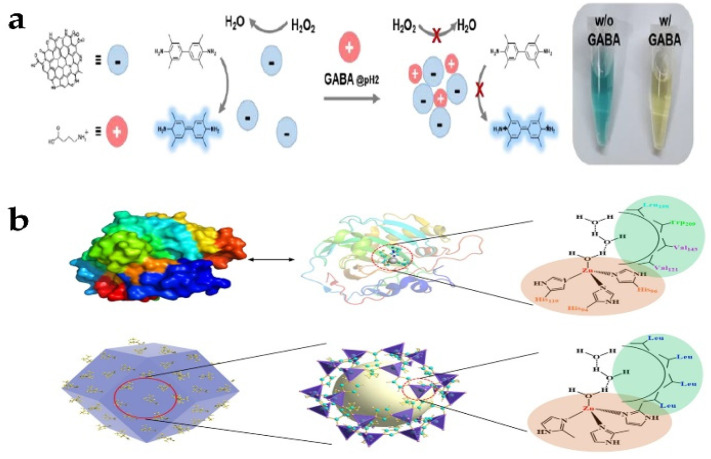
(**a**) Schematic of the peroxidase mimetic activity of Mn:N-CDs [43]. (**b**) Molecular structures of carbonic anhydrase II (CAII) and its active center as well as schematic illustration of the ZIF-8 structure and its CA-mimetic active center [44]. Reproduced with permission from (**a**) Ref. [43], Copyright 2021 Multidisciplinary Digital Publishing Institute; (**b**) Ref. [44], Copyright 2022 American Chemical Society.

Surface modifiers can be divided into three categories: ions, small molecules, and macromolecules. Lee et al. [43] introduced Mn(acetate)_2_ during the synthetic step of N-doped carbon dots to improve the enzymatic properties of metal-induced N-doped carbon dots (N-CDs). Its influence on the enzymatic properties of Mn-induced N-CDs (Mn:N-CDs) was investigated. Finally, the addition of Mn(acetate)_2_ to the reaction solution seemed to generate more functional groups at the edge of carbogenic domains in Mn:N-CDs than in N-CDs, resulting in improved peroxidase-like properties (Figure 3a). Mn:N-CDs with strong enzymatic effects can be applied as a colorimetric sensor probe for the detection of gamma-aminobutyric acid (GABA). Surface modification can also change the intrinsic enzyme activity of nanomaterials. Zeolitic imidazolate framework-8 (ZIF-8) is a monatomic nanozyme with peroxidase activity. Sun et al. [44] introduced amino acid (AA) to regulate the growth of ZIF-8 crystal, thus simulating the structure and function of natural carbonic anhydrase (CA). Amino acid as a capping agent regulates the shape and size of ZIF-8 and forms a hydrophobic region on the surface of ZIF-8 to simulate the hydrophobic pocket of natural carbonic anhydrase (Figure 3b). Compared with natural carbonic anhydrase, Val-ZIF-8 not only has excellent esterase activity but also has better hydrothermal stability. Surface coating may also weaken or even lead to loss of enzyme activity. Jain et al. [45] reported the replacement of cetyl trimithyl ammonium bromide (CTAB) by 11-MUA from the surface of Au-core CeO_2_-shell NP-based nanozyme studied for exhibiting multiple enzyme-like activities such as peroxidase, catalase, and superoxide dismutase. They found that 11-MUA coating AuNPs lost the SOD and catalase-like activity, which compromise the multifunctional property of chitosan nanoparticles (CSNPs).

### 2.4. Other Factors

**Figure 4:**
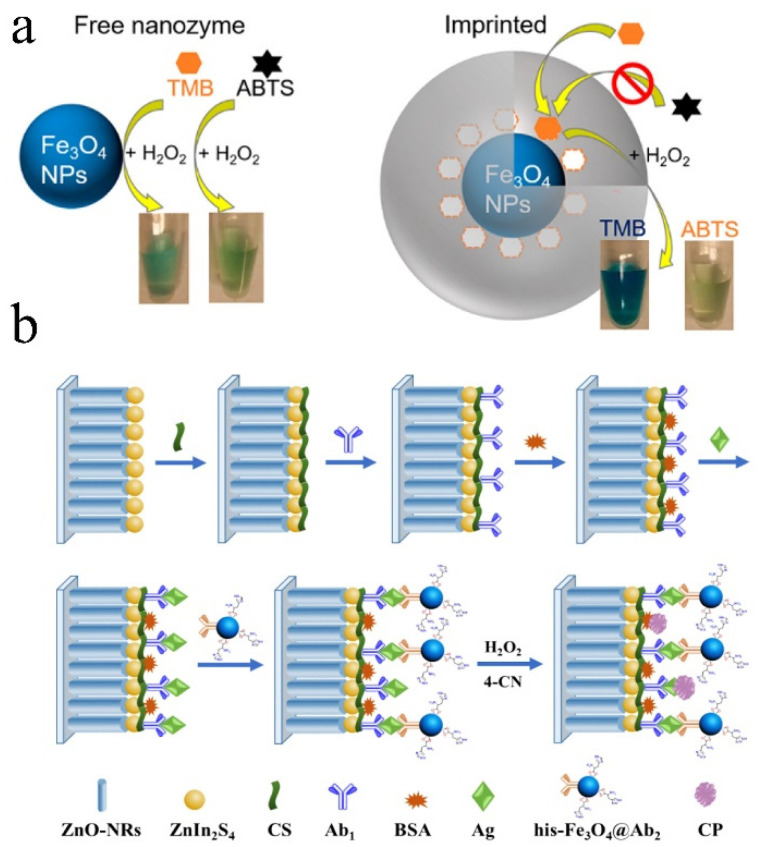
(**a**) Schematic diagram of molecularly imprinted nanozyme preparation [52]. (**b**) Schematic diagram of the PEC immunoassay using high-activity Fe_3_O_4_ nanozyme as signal amplifier. Reproduced with permission from (**a**) Ref. [52], Copyright 2017 American Chemical Society; (**b**) Ref. [53], Copyright 2019 Elsevier.

Except regulating the intrinsic enzyme activity of nanozyme to control catalytic activity, external factors can also affect the final enzyme activity. The pH and temperature are the main external influencing factors. A lot of studies have confirmed that acidic conditions are suitable for peroxidase-like activity, while neutral and alkaline conditions are favorable for superoxide dismutase and catalase. For example, the esterase activity of Val-ZIF-8 synthesized by Sun et al. [44] would greatly increase with the increase in temperature. The enzyme activity at 80 °C was about 25 times higher than that at 25 °C. Gao et al. [46] reported a new strategy for controlling plaque biofilm with a peroxidase-like nanozyme (CAT-NP). CAT-NP showed a strong dependence on acidic conditions. It killed 99% of bacteria in the acidic microenvironment simultaneously in a short time for biofilm control and prevention of dental caries. However, several studies have broken through the limitation of optimal pH for different nanozymes. Li et al. [47] developed the copper-based nanozyme (CuCo_2_S_4_), which showed enhanced peroxidase-like activity and antibacterial ability under neutral conditions (Figure 4). This would be used for infected wounds with pH close to neutral.

## 3. Improvement of the Specificity 

At present, the research of nanozyme is not only to improve catalytic activity but also to improve specific recognition ability to realize the replacement of natural enzyme. The activity change of nanozyme is related to the action mechanism of nanozyme, while the catalytic specificity of nanozyme affects the accuracy of target capture. Most applications of nanozymes are based on the discovery and simulation of nanozyme activity, but there is still a lack of catalytic specificity. The simulation of catalytic activity mostly comes from the functional replica rather than the remodeling of the active center structure of natural enzyme, so the catalytic specificity is greatly reduced. In the years of rapid development of nanozyme, some strategies have been explored to solve these problems: (I) to simulate the active center and binding site of natural enzyme more precisely from the chemical structure at the design and construction of nanozyme and (II) to combine some specific molecular-assisted recognition.

The premise for natural enzyme to work is to combine it with the substrate, that is, to capture the substrate. It mostly depends on the primary structure and spatial configuration of protein or RNA to realize the complementarity with specific substrate. Therefore, the research on the specificity of nanozyme can start from this point. Currently, there have been many reports on biomimetic research of nanozyme [48,49,50]. For example, Zhou et al. [48] proposed a chiral COF nanozyme with highly ordered active centers and substrate binding sites that is mainly used to simulate horseradish peroxidase (HRP). The active site of HRP contains porphyrin heme as the catalytic active center and the distal L-histidine (L-His) residue as the binding site. Biomimetic COF enzyme is mainly constructed by mixing iron 5, 10, 15, 20-tetra (4′-tetraphenylamino) porphyrin unit (Fe-ATPP) into the COF skeleton as the active center and modifying L-His as the substrate binding site for chiral recognition. COF can be used as the skeleton of nanozyme. The well-dispersed Fe-ATPP unit in the skeleton endows COF nanozyme with high enzyme-like activity, which is 21.7 times higher than HRP. At the same time, the incorporation of L-/D-Hiss imparts the COF nanozyme with enantioselectivity in the oxidation of L-/D-dopa enantiomers and displays a preference for dopa. Changing the content of L-/D-Hiss can also optimize the selectivity of COF nanozyme. This work can easily adjust the activity and stereospecificity of COF chiral nanozyme by changing the doped amino acid and its content.

Although the biomimetic simulation of active centers and binding sites can be carried out according to the analysis of the three-dimensional structure of natural enzymes, the biological affinity and structural simulation of nanomaterials are still limited, which still need to be assisted by biological molecules with specific recognition ability. In this method, the biological recognition element and nanozyme are coupled to achieve the dual improvement of catalytic activity and specificity. Biorecognition elements mainly include antibody, DNA, aptamer, molecularly imprinted polymer (MIP), and biological enzyme. Molecularly imprinted polymer (MIP) is a polymer processed by molecular imprinting technology, which leaves a cavity in the polymer matrix and has affinity for selected “template” molecules [51]. Molecular recognition sites of specific target molecules are created in MIP to obtain solid materials with high selectivity for specific target molecules. Zhang et al. [52] initiated polymerization on the surface of the nanozyme substrate conjugate by adding a variety of polymerization monomers, thus forming a molecular imprinted hydrogel layer. Remove the imprinted substrate molecule to obtain the substrate specific recognition site constructed on the periphery of the nanozyme (Figure 4a). In addition, the incorporation of functional monomers and charges further improved the activity and specificity of nanozyme. Under the optimum conditions, the specificity can reach 100 times. A variety of nanozyme materials, including ferric oxide, gold, cerium dioxide, and other nanomaterials with peroxidase or oxidase activity, can be significantly improved by molecular imprinting.

The biological enzyme–nanozyme cascade sensing system combined with biological enzyme is mostly used in the application of peroxide–nanozyme. Biological enzymes combine to oxidize specific substrates and produce H_2_O_2_, which in turn triggers peroxide nanozyme color or fluorescence reaction to realize signal sensing. Although biological enzymes can selectively capture target molecules, just like the reason for the birth of nanozymes, biological enzymes are limited by pH, temperature, reaction system, and other factors. At present, two kinds of enzymes are often linked on the same carrier for a cascade reaction. The ratio of enzyme content, immobilization method, and intermediate loss all affect the catalytic efficiency and sensing accuracy. The highly variable region of the antibody endows the antibody with the ability to recognize antigen specifically. Researchers often use nanozyme to replace the biological enzyme in ELISA to provide color signals. In addition to recording optical (colorimetric, fluorescent, chemiluminescence) signals for analysis, nanozyme catalysis can also trigger changes in temperature, volume, mass and pressure to form a new sensing mode. The detection methods are not confined to colorimetric detection but can also use electrochemical detection and Raman analysis. Li et al. [53] used high-activity Fe_3_O_4_ nanozyme as a signal amplifier to develop an ultrasensitive photoelectronic (PEC) immunoassay. In short, ZnO nanorods (ZnO-NRs) growing vertically on a bar indium–tin oxide (ITO) electrode were dispersed with ZnIn_2_S_4_ nanocrystals, producing a ZnIn_2_S_4_/ZnO-NRs/ITO photoelectronic as the PEC material mix to modify and capture PSA antibodies (Ab_1_). Histidine-modified Fe_3_O_4_ (His-Fe_3_O_4_) nanozyme acts as a signal amplifier and connects with the signal PSA antibody (Ab_2_) to form His-Fe_3_O_4_@Ab_2_ conjugate, which is anchored by a specific sandwich immune reaction (Figure 4b). Labeled His-Fe_3_O_4_ nanozyme as a peroxidase induced the production of insoluble and insulating precipitation, resulting in a significant reduction of photocurrent signal. Finally, the ultralow detection limit of prostate specific antigen (PSA) 18 fg/mL was achieved. Recently, the methods that can be used for the biological coupling of nanozyme and an antibody are still limited. The technology that can effectively biocouple nanozyme with an antibody or antigen is not mature [19], and the reproducibility of nanozyme-labeled immunosensor is not good, and the large-scale commercial application technology still needs to be developed.

Deoxyribonucleic acid (DNA) and aptamers are other biological recognition elements that assist nanozymes to achieve high specificity. DNA, which follows the principle of complementary base pairing and has a specific sequence, can accurately identify the target, which is widely used in biological and medical fields. However, there are some problems to be solved in the nanozyme labeled with single-stranded DNA (ssDNA). ssDNA endows the surface of nanozyme with more negative charges, which will further affect the adsorption kinetics of substrate and the catalytic activity of nanozyme. It has been found that the modification of DNA changes the surface charge state of Fe_3_O_4_NPs, thus promoting the combination of nanozyme and substrate [54]. In addition, chemical modification or physical adsorption of DNA may also block the active site of nanozyme, resulting in the reduction of catalytic efficiency. Therefore, the length, concentration, and two-dimensional structure of DNA and the surface charge distribution of nanozyme affect the catalytic activity of nanozyme [55]. Although there have been many research outputs of DNA-modified nanozymes, we still do not know enough about the mechanism of the interaction between the DNA chain on the surface of the nanozyme and the nanozyme. Most of the results are due to the trial and error of scientific researchers. Understanding the mechanism may better control the application of DNA in the field of nanozymes. The aptamer is a short DNA sequence screened in vitro, which is also highly specific. There are many aptamer sensors based on nanozyme. A simple and low-cost colorimetric analysis was established for a highly sensitive determination of Kanamycin (KAN) through integrating boron nitrate quantum dots-anchored porous CeO_2_ nanorods (BNQDs/CeO_2_) and aptamer by Zhu et al. [56] due to the large specific surface area and synergistic interaction between BNQDs and CeO_3_, which can effectively catalyze the oxidation of 3,5′,5,2′-tetramethylbenzidine (TMB). In addition, the catalytic activity of BNQDs/CeO_2_ nanozyme was significantly enhanced because of the dispersion of BNQDs/CeO_2_ nanozyme and the increase in substrate affinity after the substrate was combined with KAN-specific aptamer. KAN can combine with the aptamer to reduce the catalytic efficiency. The proposed colorimetric method realized the low detection limit of 4.6 pM. DNA and aptamer are superior to other biological recognition molecules in cost and stability, and aptamer can also further improve the detection specificity. However, there is also a complex interaction between the aptamer and the catalytic performance of nanozyme. Mechanism analysis and rule summary need to be obtained.

## 4. Environmental Monitoring

Over recent years, with the development of industry, environmental pollution has become increasingly serious, especially water pollution, which directly threatens human health. Common pollutants include heavy metal ions, dyes, phenols, pesticides, antibiotics, drugs, plasticizers, and other organic substances due to biological pollutants such as biological pathogenic bacteria or toxin pollution (Table 1). Hence, monitoring environmental pollutants has become a global concern.

### 4.1. Toxic Ions

With the rapid development of manufacturing industry around the world, overexploitation of minerals and groundwater, and industrial wastewater discharge, toxic ion pollution has become an unavoidable environmental problem. Toxic ions mainly refer to heavy metal ions, including mercury, cadmium, lead, chromium, arsenic, and other elements with potential biological toxicity. Because it cannot be degraded, it can only be converted into different chemicals through abiotic or biological mechanisms and can be amplified through the food chain, posing a serious threat to the ecosystem and human health [57]. At present, many methods based on nanozyme detection of toxic ions have been explored. Most of the sensors are miniaturized and portable to be used in point-of-care testing (POCT).

**Figure 5:**
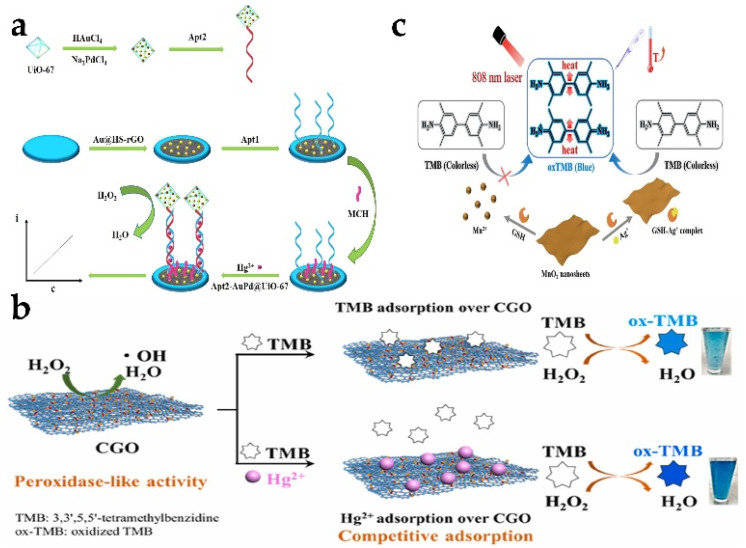
(**a**) Schematic diagram for fabricating the designed electrochemical aptasensor to detect Hg^+^ [58]. (**b**) Schematic of CGO preparation and the principle of color rendering [59]. (**c**) Schematic of the proposed MnO_2_ NSs-GSH-TMB colorimetric and photothermal platform for the Ag^+^ analysis [61]. Reproduced with permission from (**a**) Ref. [58], Copyright 2022 Elsevier; (**b**) Ref. [59], Copyright 2022 Elsevier; (**c**) Ref. [61], Copyright 2019 Springer.

Wang et al. [58] loaded Au NPs onto HS-rGO to modify a glass carbon electrode (GCE) as a sensing platform. Au Pd-modified zirconium metal organic skeleton (AuPd@UiO-67) labeled with signal chain (Apt2) is used as signal enhancer to capture Hg^2+^ based on T-Hg (II)-T structure (Figure 5a). With the increase in modified Hg^2+^ concentration, the amount of Apt2-AuPd@UiO-67 is increased, thus realizing the detection of Hg^2+^. The electrochemical sensor has a wide linear range (1.0 nmol/L–1.0 mmol/L) and a low detection limit (0.16 nmol/L). Except for electrochemical detection methods, colorimetry is the most commonly used method to detect Hg^2+^. A graphene oxide nanosheet (CGO) based on L-cysteine functionalization was found to have a strong peroxidase-like activity compared with graphene oxide [59]. The introduction of more S and N species can effectively produce more surface defects and active sites, thus endowing carbon high peroxidase-like properties. The nanozyme can be used to realize the microdetection of Hg^2+^, and its sensing principle is mainly based on the competitive adsorption between Hg^2+^ and photothermal properties of 3,3′,5,5′-tetramethylbenzidine (TMB). Because Hg^2+^ hinders the combination of TMB and CGO, TMB is catalyzed by H_2_O_2_ to produce more colored oxidation products, resulting in a more significant colorimetric response (Figure 5b), meaning, therefore, good detection of Hg^2+^. Zhong et al. [60] used the peroxidase-like activity of iron hydroxide (FeOOH) nanorods to detect As(V) by colorimetry. Unlike TMB, the catalytic substrate is ABST, and its oxidation product is green and reaches the maximum absorption peak at 418 nm. As(V) can be adsorbed onto FeOOH nanorods through electrostatic interaction and an As-O bond, so the oxidation is gradually embedded. Finally, the colorimetric determination with response of 0–8 ppb and 8–200 ppb and detection limit of 0.1 ppb is realized. Ag^+^ is also a heavy metal ion. Zhang et al. [61] utilized the excellent colorimetric and TMB to construct photothermal and colorimetric double-readout sensors for Ag+ analysis. MnO_2_ nanosheets (NSs) were used to catalyze the oxidation of TMB to oxTMB. However, the reduction of MnO_2_ NSs by glutathione (GSH) can reduce the catalytic capacity of MnO_2_ NSs (Figure 5c). In this method, a specific combination of Ag^+^ and GSH is utilized to inhibit this reduction process. According to this principle, the Ag^+^ concentration can be converted into temperature and color signals. Consequently, the Ag^+^ content can be determined both with the naked eye and with a portable thermometer. It is very suitable for POCT in the process of environmental detection.

### 4.2. Organic Pollutants

Herbicides and pesticides used in agriculture to increase food production will cause serious pollution of soil and water quality. At present, most pesticides on the market are not degradable organophosphorus pesticides. If the metabolites or degradation products of pesticides exceed the maximum residue, they will cause pollution. In addition, antibiotic residues are also considered as typical organic pollution. Because of their longer half-life, they are more threatening to humans and other organisms. Another common organic pollutant, phenolic compounds, are common in dye, pharmaceutical, photo development and other industrial fields and is difficult to degrade in the aquatic ecological environment.

**Figure 6:**
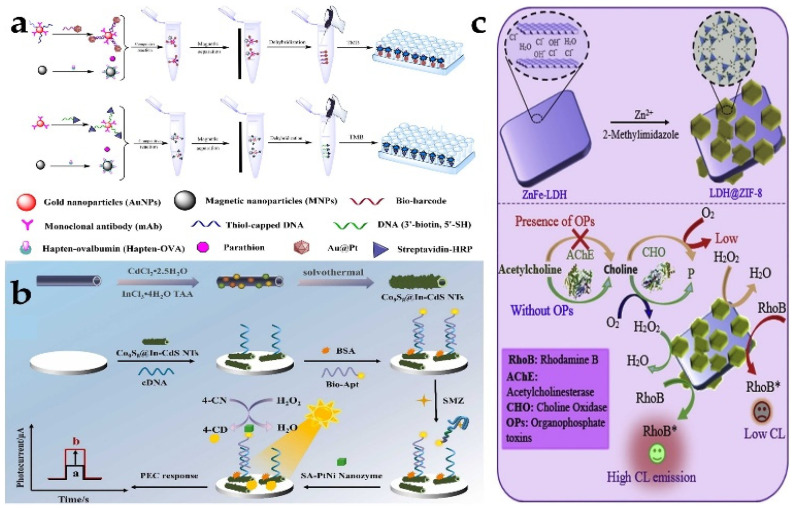
(**a**) Schematic presentation of the colorimetric biobarcode immunoassays for parathion based on amplification using Au@Pt and HRP [62]. (**b**) Schematic illustration of the preparation process of Co9S8@In-CdS NTs and the proposed PEC aptasensor for SMZ detection [64]. (**c**) Detection of OPs by LDH@ZIF-8-assisted RhoB-H_2_O_2_ CL-based system [63]. Reproduced with permission from (**a**) Ref. [62], Copyright 2020 American Chemical Society; (**b**) Ref. [64] Copyright 2022 Elsevier; (**c**) Ref. [63], Copyright 2019 Elsevier.

Parathion is an organophosphate (OP) insecticide, and it is also an irreversible inhibitor of nervous system function. Chen et al. [62] designed a bimetallic nanozyme (Au@Pt: gold@platinum) catalytic competitive sensitive biological bar code immunoassay. This novel biobarcode immunoassay contained three types of probes: (I) mAbs and ssDNA-labeled AuNP probes, (II) parathion OVA-hapten-modified immunomagnetic nanoparticle (MNP) probes, and (III) C-ssDNA-labeled Au@Pt probes (Figure 6a). The Au@Pt probe reacts with the AuNP probe through complementary base pairing. Parathion then competes with the MNP probe to bind mAb onto the AuNP probe. Finally, Au@Pt nanozyme is released from the complex to catalyze the color development of TMB. The most common and efficient platform to detect OPs is the enzyme biosensor, which is based on OP_S_ to inhibit acetylcholinesterase (AChE) activity. In another work, lactate dehydrogenase (LDH)-based ZIF-8 nanocomposite was prepared by Bagheria et al. [63], utilizing the formation of a simple complex between Zn^2+^ and 2-methylimidazole. This process leads to the formation of highly dispersed ZIF-8 nanostructures on the surface of ZnFe-LDH nanosheets (LDH@ZIF-8). Moreover, the peroxidase-mimicking behavior of the prepared nanocomposites is improved compared to pristine LDHs and MOFs. LDH@ZIF-8 significantly contributes to the CL emission intensity of the H_2_O_2_ rhodamine B (RhoB) system. When organophosphorus pesticides (OP_S_) were present, AChE activity was inhibited, and reduced production of H_2_O_2_ eventually led to the attenuated chemiluminescence of RhoB (Figure 6c). This detection method achieved highly sensitive sensing of OP_S_ indirectly by detecting the change of the hydrogen peroxide content. Apart from the chemiluminescence strategy, photoelectrochemical biosensors are another emerging analytical method. Song et al. [64] used the double amplification strategy to construct the photoelectrochemical aptamer sensor. The sensor realizes signal sensing mainly based on Co_9_S_8_@In-CdS NTs and PtNi nanozymes acting as signal amplifiers. Because sulfamethazine (SMZ) has a stronger affinity with the aptamer, the aptamer tends to combine with SMZ and escape from the electrode through the dissociation of the double chain structure, generating a photocurrent response. SA-modified PtNi nanostructures further increase the spatial steric resistance of the electrode surface. The insoluble 4-CD precipitate formed by incubation with 4-CN in the presence of H_2_O_2_ seriously hinders electron transfer and again changes the photocurrent response to achieve sensing of SMZ (Figure 6b).

In order to make detection and environmental assessment portable and fast, some researchers bind sensors and smartphones to achieve mobile data transmission. This kind of combination can be used for POCT. Sun et al. [65] synthesized a three-layer FeOx@ZnMnFeOy @Fe Mn bimetallic organism with excellent multienzyme activity (peroxidase, oxidase, and catalase). Therefore, this sensing platform can complete the four functions of detecting H_2_O_2_, citric acid (CA), norfloxacin (NOR), and gallic acid. Moreover, smartphones are also used for automatic quantitative detection of CA and NOR, and the detection limit of norfloxacin is as low as 52 nM. Nanozyme with multienzyme activity was also used in the detection of phenolic compounds. A highly efficient mimic catalyst of Co_1.5_Mn_1.5_O_4_ with four enzyme activities (peroxidase, oxidase, catalase, laccase) was used to detect dihydroxybenzene isomers. Finally, a dual function colorimetric sensor was constructed using TMB [66]. Ye et al. [67] constructed NiCo_2_O_4_@MnO_2_ with p–n junctions, which not only has the photoelectric effect brought by the p–n junctions but also has inherent oxidase and peroxidase-like activities. The result is an excellent minimum detection limit of 0.0042 μM for hydroquinone.

### 4.3. Foodborne Pathogens

Biological pollution mainly refers to environmental pollution caused by various organisms that pose a threat to human health. The biological pollution in the water and soil environment mainly comes from untreated domestic sewage, industrial wastewater, garbage, and feces, which eventually leads to the excessive content of foodborne pathogens. In history, Vibrio cholerae once polluted the water environment as a foodborne pathogen, which eventually led to the global epidemic of cholera in the 1930s. Intestinal bacteria such as Escherichia coli, Streptococcus faecalis and Clostridium are the main bacteria that pollute water. Hepatitis also erupts through fecal sewage. 

**Figure 7:**
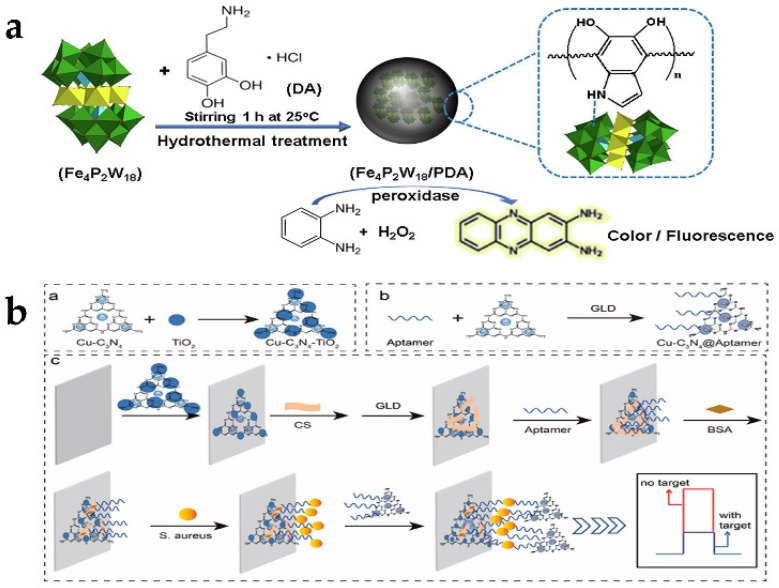
(**a**) Construction of the PEC sensor and detection of S. aureus [72]. (**b**) Schematic illustration of the synthesis procedure and the peroxidase activity of the P2W18Fe4/PDA nanozyme [71]. Reproduced with permission from (**a**) Ref. [72], Copyright 2022 Elsevier; (**b**) Ref. [71], Copyright 2021 Elsevier.

The first microorganism to be detected in drinking water is the content of Escherichia coli (*E. coli*). Thus, the detection of foodborne pathogens is increasingly urgent. In the detection of *E. coli*, β-Galactosidase (β-Gal) is applied. A multicolor colorimetric platform triggered by a designed enzyme nanozyme cascade reaction was designed and prepared [68]. MnO_2_ nanoparticles with oxidase-like activity can catalyze the oxidation of TMB. Then TMB^2+^ quickly etched the gold nanorods (Au NRs), and the longitudinal local surface plasmon resonance peak appeared as an obvious blue shift and the polychromatic change of Au NRs. The presence of *E. coli* will hydrolyze p-aminophenyl β-d-galactopyranoside (PAPG) to produce p-aminophenol (PAP) through β-galactose, thereby mediating the reduction of MnO_2_ nanosheets, destroying their oxidase-mimicking activity, and affecting the production of TMB^2+^. Consequently, sensing systems that exhibit different colors can be easily observed for different concentrations of *E. coli*. A colorimetric sensor based on Ps-Pt nanozymes for the detection of Salmonella typhimurium was reported by Jiao et al. [69]. The sensor combines immunosensing and magnetic separation techniques. They covalently bound streptavidin first to Ps-Pt. Then the bacteria were recognized by coupling a biotinylated antibody of *S. typhi* onto PS Pt through the high affinity between streptavidin and biotin. Moreover, the magnetic beads conjugated antibodies were also prepared to facilitate the subsequent bacteria separation test, thus making the detection simple and fast. Similarly, Ps-Pt nanozyme with peroxidase activity has also been used to detect *Staphylococcus aureus* (*S. aureus*) on paper-based analytical equipment with a detection limit of 9.56 ng/mL [70]. Targeting *S. aureus*, Luo et al. [71] constructed a PEC sensor for *S. aureus* with a wide linear range between 10 and 10^8^ CFU/mL and a limit of detection (LOD) as low as 3.40 CFU/mL based on “signal off” using the Cu-C_3_N_4_-TIO_2_ heterostructure as the photoactive material and Cu-C_3_N_4_ peroxidase-like nanozymes as signal amplifiers. During the detection, Cu-C_3_N_4_ (Cu-C_3_N_4_@Apt) and benzo-4-chlorohexanedione (4-CD) produced by the oxidation of 4-chloro-1-naphthol (4-CN) in the presence of hydrogen peroxide participated in decreasing the photocurrent signal (Figure 7b). Polyoxometalates (POMS) of different structures have also been noted to possess peroxidase activity, among which P_2_Fe_4_W_18_ enzyme activity was reported by Zhang et al. [72] Polydopamine (PDA) as an emerging biomimetic adhesive polymer combines with P_2_Fe_4_W_18_ to enhance enzyme activity (Figure 7a). Ultimately, Fe_4_P_2_W_18/_PDA achieved the detection of *E. coli* O157:H7 with a detection limit of 4.2 × 10^2^ CFU/mL.

**Table 1:** Summary of the application of nanozymes in environmental monitoring.

Category	Analyte	Nanozyme	Activity	Detection Mode	Detection Range	LOD	Ref.
Toxic ions	Fe^2+^/Pb^2+^	MnO_2_	CAT	Colorimetric	0.001~0.02 mmol/L 0.05~0.4 mmol/L	0.5 μmol/L 2 μmol/L	[73]
	F^−^	AgPt-Fe_3_O_4_	POD	Colorimetric	50~2000 μM	13.73 μM	[74]
	Nitrite	AuNP-CeO_2_ NP@GO	OXD	Colorimetric	100~5000 μM	4.6 M	[75]
	Cl^−^, Br^−^, I^−^	Ag_3_Cit	OXD	Colorimetric	/	26, 12, 7 nM	[76]
	Cu^2+^	E-ChlCu/ZnO	POD	Colorimetric	0–1/1–15 μM	0.024 μM	[77]
	As^3+^	Pd-DTT	OXD	Colorimetric	33~3.333 × 10^5^ ng/L	35 ng/L	[78]
	Fe^2+^	C-dots/Mn_3_O_4_ NCs	OXD	Colorimetric	0.03~0.83 μM	0.03 μM	[79]
	Nitrite	His@AuNCs/RGO	POD	Electrochemical	2.5~5700 μM	0.5 μM	[80]
	Hg^2+^	MXene/DNA/Pt NCs	POD	Colorimetric	50~250 nM	9.0 nM	[81]
	Fe^3+^	NCD/UiO-66 NCs	SOD POD	Colorimetric	0~0.1 mM	/	[82]
	Cr^6+^	PEI-AgNCs	OXD	Colorimetric	/	1.1 μM	[83]
	Fe^2+^	AuRu aerogels	OXD POD	Colorimetric	5~250 μmol/L	0.7 μmol/L	[84]
	Hg^2+^	CS-MoSe_2_NS	POD OXD	Colorimetric	0.1~4.0 μM	3.5 nM	[85]
	Fe^3+^	MoSe_2_@Fe	POD	Colorimetric	25~300 μM	1.97 μM	[86]
	F^−^	R-MnCo_2_O_4_/Au NTs	POD	SERS	0.1~10 nM	0.1 nM	[87]
	Sn^2+^	nano-UO_2_	POD	Colorimetric	0.5–100 μM	0.36 μM	[88]
	PO_4_^3−^	MB@ZrHCF	POD	Colorimetric	10~200 μM	2.25 μM	[89]
	Cr^3+^	GdOOH	Phospholipase	Colorimetric	5.0~200 μM	0.84 μM	[90]
	Hg^2+^	AuPd@UiO-67	POD	Electrochemical	1~10^6^ mM	0.16 nmol/l	[58]
	Al^3+^	Single atom Ce-N-C	Laccase	Colorimetric	5–25 μg/mL	22.89 ng/mL	[91]
	Cr^6+^	CD/g-C_3_N_4_	POD	Colorimetric	0.3–1.5 μM	0.31 μM	[92]
	Hg^2+^	CuS HNS	POD	Colorimetric	50~4 × 10^5^ ng/mL	50 ng/L	[93]
	As^3+^	CoOOH	POD	Electrochemical	0.1~200 μg/L	56.1 ng/L	[94]
	Cr^6+^	Cu-PyC MOF	POD	Colorimetric	0.5–50 μM	0.051 μM	[95]
	Cr^6+^	Ni/Al LDH (Ni/Al–Fe(CN)_6_ LDH)	POD	Colorimetric	0.067~10 mM	0.039 mM	[96]
	Pb^2+^	Tannic Acid@Au NPs	POD	Colorimetric	25~500 ng/mL	11.3 ng/mL	[97]
	S^2−^	MoS_2_/g-C_3_N_4_HNs	POD	Colorimetric	0.1~10 μM	37 nM	[98]
	S^2−^	PDA@Co_3_O_4_NPs	CAT	Colorimetric	4.3~200 μM	4.3 μM	[99]
	As^3+^	AuNPs	POD	Colorimetric	0.01~11.67 mg/L	0.008 mg/L	[100]
	S^2−^	GMP-Cu	Laccase	Colorimetric	0~220 μmol/L	0.67 μmol/L	[101]
	Hg^2+^	Ag_2_S@GO	OXD	Colorimetric	5.0~120.0 × 10^−8^ M	9.8 × 10^–9^ mol/L	[102]
	Cu^2+^	MMoO	POD	Colorimetric	0.1~24 μM	0.024 μM	[103]
	Cr^6+^	MOF	OXD	Colorimetric	0.1~30 μM	20 nM	[104]
	Cr^6+^	CuS-frGO	POD	Colorimetric	0–200 nM	26.60 nM	[105]
	Cr^6+^	SA-Fe/NG	POD	Colorimetric	30~3 μM	3 nM	[106]
	Cr^3+^	CuFe_2_O_4_/rGO	POD	Colorimetric	0.1~25 μM	35 nM	[107]
	Hg^2+^	L-cysteine@GO	POD	Colorimetric	0~200 μg/L	5 μg/L	[59]
	Hg^2+^	PtNPs	POD	Colorimetric	20~3000 nM	10.5 nM	[108]
	Hg^2+^	Au-HBNz	POD	Colorimetric	0.008~20 μg/mL	1.10 ng/mL	[109]
	Hg^2+^	AuPt@DSN	POD	Colorimetric	0.1~10^3^ nM	8.58 pM	[110]
	Hg^2+^	MVC-MOF	OXD	Colorimetric	0.05~6 μM	10.5 nM	[111]
	Hg^2+^	Citrate-capped Cu NPs	POD	Colorimetric	0.100~6.000 μM	0.052 μM	[112]
	Hg^2+^	Fe-MoS_2_@AuNPs	POD	Electrochemical	0.5~200 nM	0.2 nM	[113]
	Hg^2+^	Ag NWs	OXD	Colorimetric	25∼5000 μg/L	19.9 ng/L	[114]
	Hg^2+^	Cys-Fe_3_O_4_	POD	Colorimetric	0.02–90 nM	5.9 pM	[115]
	Hg^2+^	His-AuNCs	OXD	Colorimetric	0.05–0.8 μM	8 nM	[116]
	Ag^+^	MnO_2_ NSs	OXD	Colorimetric	0.02~1.0 μM	6.7 nM	[61]
	As^5+^	FeOOH	POD	Electrochemical	0.04~200 μg/L	12 ng/L	[60]
	Al^3+^	Nanoceria	Phosphatase	Electrochemical	30~3.5 × 10^3^ nM	10 nM	[117]
	H_2_O_2_	MA-Hem/Au-Ag	POD	Colorimetric	0.010–2.50 mM	2.5 μM	[118]
	H_2_O_2_	Pt/CeO_2_/NCNFs	CAT	Electrochemical	0.0005–15 mM	0.049 μM	[119]
Phenolic	Phenol Compounds	1-Methylimidazole/Cu Nanozyme	Laccase	Colorimetric	0.5~4 μg/mL	0.57 μg/ml	[120]
	2,4-dinitrophenol	polymer-Fe-doped ceria/Au NC	POD	Colorimetric	1~100 μg/mL	2.4 μM	[121]
	Hydroquinone	NiCo_2_O_4_@MnO_2_	POD OXD	Colorimetric	0~24 μM	0.042 μM	[67]
	Hydroquinone	Co_1.5_Mn_1.5_O_4_	OXD	Colorimetric	0.05∼100μM	0.04μM	[66]
	2,4,6-TNT	2H–MoS_2_/Co_3_O_4_	OXD	Electrochemical	/	1 pM	[122]
	Hydroqui-none	Fe_3_O_4_@COF	POD	Colorimetric	0.5~300 μmol L	0.12 μmol L	[123]
	2,4-DP	AMP-Cu	Laccase	Colorimetric	0.1~100 μmol/L	0.033 μmol/L	[124]
	2,4-DP	MnCo@C NCs	Laccase	Electrochemical	3.1~122.7 μM	0.76 μM	[125]
	2,4-DP	NiFe_2_O_4_	POD	Colorimetric	0.218~3.282 μg/mL	0.311 μg/mL	[126]
OPs	Carbendazim	MoS_2_/MWCNTs	OXD	Electrochemical	0.04~100 μM	7.4 nM	[127]
	parathion ethyl	C-Au NPs	POD	Colorimetric	11.6~92.8 ng/mL	5.8 ng/mL	[128]
	Dichlorvos	γ-MnOOH NWs	OXD	Colorimetric	0~15 ng/mL	3 ng/ml	[129]
	Diazinon	LDH@ZIF-8	POD	Colorimetric	0.5~300 nM	0.22 nM	[63]
	Paraoxon	2D MnO_2_	OXD POD	Electrochemical	0.1~20 ng/mL	0.025 ng/mL	[130]
	Benomyl	AgNPs/MWCNTs/GO	OXD	Electrochemical	0.2~122.2 μM	/	[131]
	Dimethoate	Pt NPs	POD	Colorimetric	0.5~9 μg/mL	0.15 μg/mL	[132]
	Naphthalene acetic acid	Ti_3_C_2_-MXene/BP	OXD	Electrochemical	0.02~40 μM	1.6 nM	[133]
	Parathion	NiO-SPE	OXD	Electrochemical	0.1~30 μM	0.024 μM	[134]
	MeHg	NA-CDs/AuNPs	POD	Colorimetric	0.375~75 μg L	0.06 μg L	[135]
	Chlorpyrifos	Ag-Nanozyme	POD	Colorimetric	35~210 ppm	11.3 ppm	[136]
	Omethoate	SACe-N-C	POD	Colorimetric	100~700 μg/mL	55.83 ng/mL	[137]
	Methyl-paraoxon	Nanoceria	Laccase	Colorimetric	0.42~126 μM	0.42 μmol/L	[138]
	Methyl-paraoxon	CeO_2_	POD OXD	Electrochemical	0.1~100 μmol/L	0.06 μmol/L	[139]
	Methyl-parathion	Fe_3_O_4_/C-dots@Ag-MOFs	/	Electrochemical	5 × 10^−11^~2 × 10^−9^ mol/L	1.16 × 10^−11^ mol/L	[140]
	Atrazine	Fe_3_O_4_-TiO_2_/rGO	POD	Colorimetric	2~20 mμ g/L	2.98 μg/L	[141]
	Glyphosate	Au@PN	POD	Colorimetric	0.5~20 nM	0.24 nM	[142]
	Glyphosate	Porous Co_3_O_4_	POD	Colorimetric	8~80 μg/L	2.37 μg/L	[143]
	Glyphosate	Fe_3_O_4_@C_7_/PB	POD	Colorimetric	0.125~15 μg/mL	0.1 μg/mL	[144]
	Carbaryl	NH_2_-MIL-101(Fe)	POD	Colorimetric	2~100 ng/mL	1.45 ng/mL	[145]
	Chlorophenols	Fe_3_O_4_@MnOx	OXD	Colorimetric	10~1600 μM	0.85 μM	[146]
	Fipronil	ZIF-8	POD	Colorimetric	0.2~4μM	0.036 μM	[147]
	Malathion	Fe-N/C SAzyme	OXD	Colorimetric	0.5~10 nM	0.42 nM	[148]
Antibiotic residues	Sulfamethazine	PtNi NCs	POD	Photoelectrochemical	0.05~10^3^ pg/mL	37.2 fg/mL	[64]
	Sulfonamides	2D Cu-TCPP (Fe)	POD	Electrochemical	1.186~28.051 ng/mL	0.395 ng/mL	[149]
	Streptomycin	Au@Pt NPs	POD	Lateral Flow Immunoassays	0.062~0.271 ng/mL	1 ng/mL	[150]
	Tetracycline	Cu-doped-g-C_3_N_4_	POD	Colorimetric	0.1~50 μM	31.51 nM	[151]
	Tetracycline	Fe_3_O_4_@MIP	POD	Colorimetric	2~225 μM	0.4 μM	[152]
	Tetracycline	MIL-101(Fe/Co)	POD	Colorimetric	1–8 μM	0.24 μM	[153]
	Norfloxacin	FO@ZMFO@FM-MOG	CAT OXD POD	Colorimetric	0.415–6.21 μM	52 nM	[65]
	Kanamycin	CoFe_2_O_4_NPs	POD	Electrochemical	1~10^−6^ μM	0.5 pM	[62]
	Chloramphenicol	Co_3_O_4_	POD	Electrochemiluminescence	5 × 10^−13^~4 × 10^−10^ mol/L	1.18 × 10^−13^ mol/L	[154]
	Kanamycin	WS_2_ Nanosheets	POD	Colorimetric	0.1–0.5 μM	0.06 μM	[155]
	Metronidazole	MIL-53 (Fe)@ molecularly imprinted polymer (MIP)	POD	Colorimetric	1~200 μM	53.4 nM	[156]
Foodborne pathogens	*Staphylococcus aureus*	Cu-C_3_N_4_-TiO_2_	POD	Photoelectrochemical	10~10^8^ CFU/mL	3.40 CFU/mL	[71]
	*Staphylococcus aureus*	Pd@Pt NPs	POD	Lateral Flow Immunoassays	10–300 ng/mL	9.56 ng/mL	[70]
	*Salmonella typhimurium*	IPs-Pt	POD	Colorimetric	10^4^~10^6^ CFU/mL	10^3^ CFU/mL	[69]
	*Escherichia coli*	Au NRs	OXD	Colorimetric	1.0 × 10^2^~1.0 × 10^5^ CFU/mL	22 CFU/mL	[68]
	*E. coli* O157:H7	Au-Pt dumbbell NPs	POD	Colorimetric	10~10^7^ CFU/mL	2 CFU/mL	[157]
	*E. coli* O157:H7	man-Pediatric lead (PB)	POD	Lateral Flow Immunoassays	10^2^~10^8^ CFU/mL	10^2^ CFU/mL	[158]
	*E. coli* O157:H7	P_2_W_18_Fe_4_/PDA	POD	Colorimetric	10^3^~10^6^ CFU/mL	4.2 × 10^2^ CFU/mL	[72]

## 5. Environmental Management

Over these years, industrial development and natural resource exploitation have brought economic prosperity, but at the same time, all over the world, it has also faced serious environmental governance challenges. Most pollutant residues are found in water sources and soils on which mankind depends for survival. Furthermore, the common degradation methods of pollutants are of three types: (I) biodegradation, (II) physical adsorption, and (III) chemical oxidation. Nanozymes as an emerging research outcome in the 21st century exhibit excellent qualities in environmental governance. They (I) can handle compounds that are often difficult to biodegrade, (II) can operate independently of pollutant concentration, (III) can operate over a wide range of pH, temperature, and salinity, (IV) are not inhibited by biofouling, (V) are relatively simple and easy to control, and (VI) are highly stable and recyclable [57]. Environmental monitoring Table 2 demonstrates the application of nanomaterials in the degradation of various pollutants.

Currently, most of the substances that nanozymes are able to degrade are organic such as phenolic compounds, herbicides, insecticides, dyes, and antibiotics. A few nanozymes are used to degrade inorganic heavy metal ions. Citric acid-modified copper peroxide nanodots (CP@CA) synthesized by Yang et al. [182] are an autocatalytic nanozyme. Under acidic conditions, they can decompose into H_2_O_2_ and Cu^2+^ in water or soil, while H_2_O_2_ would further decompose into •OH, capable of degrading nicosulfuron, based on a Fenton-like reaction. Its degradation rate can reach 97.58% within 1 h. Furthermore, after CP@CA was involved in pollutant degradation, the ecotoxicity of most degradation intermediates was reduced to a lower level compared with nicosulfuron. Moreover, CP@CA had little effect on the active components of the soil bacterial community. Photocatalytic degradation is another pathway for the degradation of pollutants. Baruah et al. synthesized magnetic Fe_3_O_4_ NPs on the surface of polydopamine functionalized RGO sheets (FDGs) for photocatalytic degradation of the hazardous pesticide simazine. Due to its excellent photocatalytic activity and magnetic separability, this makes the degradation rate of this nanozyme 99% and highly sustainable. The specific mechanism of its degradation relies on the formation of •OH under photocatalysis. The graphene sheets with good optical properties enhanced Fe_3_O_4_ with very high electron hole pair recombination characteristics. Dopamine-functionalized graphene sheets (DG) have high electron carrier capacity through their π-bond network, resulting in FDG nanozymes with high photocatalytic activity. Upon light irradiation, nanozymes absorb photons and undergo redox reactions by elevating electrons from the valence band (VB) to the conduction band (CB) (Eq^n^ 8). The electrons in the CB can be easily transferred to the DG surface, forming a hole in the VB (Eq^n^ 8). The electrons on the surface of DG simultaneously trap dissolved molecular oxygen and lead to the formation of superoxide radical anions (O•^2−^) (Eq^n^ 9). Superoxide radical anions directly interact with water molecules to form •OH (Eq^n^ 10–12). Similarly, the holes (H^+^) can be in contact with water molecules producing •OH (Eq^n^ 13). •OH decomposes simazine pesticides into nontoxic inorganic molecules and ions [181].

**Figure 8:**
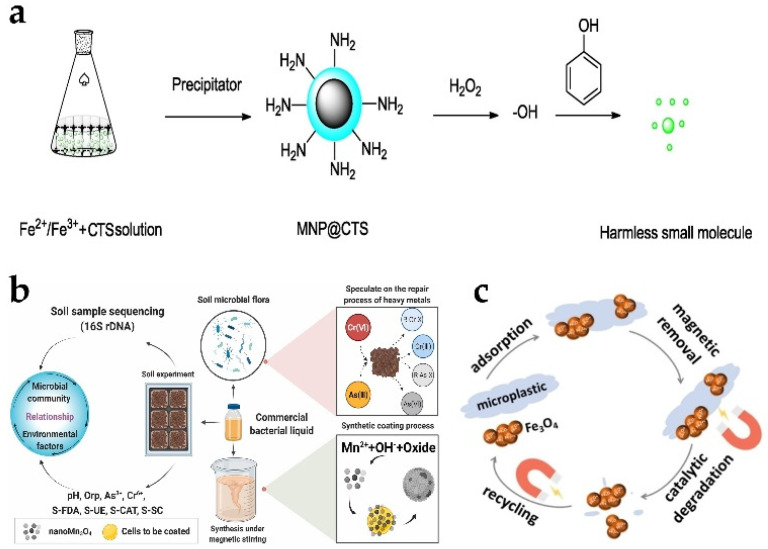
(**a**) Schematic diagram of MNP@CTS nanozyme-catalyzed degradation of phenol [171]. (**b**) Experimental design and process on NMCMP (NMCMP: nanozyme nanoMn_3_O_4_-coated microbial populations) [167]. (**c**) Schematic diagram of Fe_3_O_4_ nanozyme-catalyzed degradation of microplastics [177]. Reproduced with permission from (**a**) Ref. [171], Copyright 2018 Elsevier; (**b**) Ref. [167], Copyright 2022 Elsevier; (**c**) Ref. [177], Copyright 2022 Wiley Online Library.

There are few recent studies on the removal of heavy metal ions by nanozyme, but there are still several methods with high removal efficiency. AgRu bimetal mesoporous nanozyme costabilized by β-CD and GO (AgRu@ β-CD co GO) was first constructed [168]. The nanozyme has a porous microstructure and a large number of hydroxyl and GO aromatic rings, which can enrich and adsorb a large amount of Hg^+^ and Cl^−^ in water. The authors, through a 0.22 μm commercial millipore filtration membrane, repeatedly filtered the mixed solution containing Hg^+^, Cl^−^, and nanozyme three times, and the Hg2^+^ and Cl^−^ removal efficiency reached more than 95.4% and 93.8%, respectively. In another work, Su et al. [167] investigated the microbial sensitivity regulation mechanism (MSRM) on typical paddy field heavy metal pollution (As^3+^ and Cr^6+^) using nanozyme nanoMn_3_O_4_-coated microbial populations (NMCMP) and proved that Flavoisolibacter and Arthrobacter were two main bacteria related to heavy metal (As^3+^ and Cr^6+^) pollution remediation. In addition, NMCMP can enhance the reduction of Cr^6+^ level and inhibit the release and rapid oxidation of As^3+^ during the repair process of As_2_H_2_S_3_ (Figure 8b). Methyl orange is a typical dye in industrial wastewater selected as a typical dye pollutant because it is not easily degraded. CNZ was applied to the degradation of methyl orange pollutants [160]. At a high temperature of 60 °C and pH value of 3.93%, the degradation rate can be obtained in less than 10 minutes. In addition, the nanozyme showed excellent reusability and storage stability. However, Pd@ZnNi-MOF/GO nanocomposites with high peroxidase-like activity took only 8 min to completely degrade the methyl blue dye, and the catalytic degradation efficiency was as high as 95% [162].

Decomposition of phenol and phenolic compound purification of the environment is the focus of social attention. The degradation of phenol and phenolic compounds using ferromagnetic nanoparticles (MNPs) has many advantages. Ferromagnetic chitosan nanozymes (MNP@CTS) have the ability to catalyze the production of reactive oxygen species from hydrogen peroxide. Under the action of reactive oxygen species, the substrate phenol can be rapidly oxidized into various small molecules. Meanwhile, CTS can improve the catalytic efficiency and increase the degradation rate and degradation effect (Figure 8a). The removal efficiency is higher than 95% within 5 h [171]. Microplastics have a high surface-to-volume ratio, and they can act as a carrier for invading microorganisms, heavy metals, and other contaminants. Some of the long-term deleterious effects of microplastics include infertility, degradation of microplastics, and cancer. Therefore, it is crucial to remove and degrade microplastics in water resources. Hydrophilic bare Fe_3_O_4_ nanoaggregates allowed efficient removal of the most common microplastics including high-density polyethylene, polypropylene, polyvinyl chloride, polystyrene, and polyethylene terephthalate [177]. The bare Fe_3_O_4_ nanoaggregates with peroxidase-like activity further catalyzed the degradation of microplastics with nearly 100% efficiency by adsorbing to microplastics via hydrogen bonding (Figure 8c).

## 6. Other Environmental Protection Applications

### 6.1. Air Purification

Air pollution does great harm to people who breathe with their lungs. Formaldehyde is particularly harmful. Newly decorated rooms often face the problem of too much formaldehyde and cannot be occupied. Ecological nanozymes can catalyze the decomposition of formaldehyde. The average purification rate of formaldehyde in two hours was 91.9% [57]. The composite material is made of activated carbon fiber (ACF) and porous polymer composite and is also equipped with an antibacterial agent. When formaldehyde molecules are sucked into the nanospace, the nanozyme will react with the oxygen in the air to produce highly active superoxide ions with active oxygen structure. Due to the large contact area between the ecological enzyme catalyst and the adsorbed formaldehyde molecules in the nanopores, the active catalyst molecules quickly combine with formaldehyde molecules. After a series of oxidation-reduction enzyme catalyzed reactions, different peroxyintermediate oxidation molecules are formed. Finally, the formaldehyde molecules are oxidized into water and carbon dioxide molecules [57]. However, the ecological enzyme quickly returned to its original state and combined with oxygen molecules in the air again. The process of “combination with oxygen molecules– formation of active oxygen molecules–combination with formaldehyde molecules–enzymatic decomposition” keeps repeating so as to remove formaldehyde, bacteria, and other organic molecules in the air. This can keep the composite material clean for a long time.

### 6.2. Antibacterial and Antifouling Agent

Marine biofouling refers to the process in which marine microorganisms, animals, and plants continuously enrich and grow on artificial surfaces to form biofouling, which is a worldwide problem affecting maritime transport and communication facilities and coastal power plants [183]. The microfouling organisms on the substrate surface form a heterogeneous biofilm, which is composed of a variety of heterotrophic bacteria, cyanobacteria, diatoms, protozoa, and fungi [184]. Biofouling can increase hull roughness and weight, increase navigation resistance, greatly increase fuel consumption, cause economic losses of billions of dollars every year, increase carbon dioxide emissions, and intensify the room temperature effect [185]. In addition, organisms attached to distant ships will enter different sea areas, causing potential “species invasion” and affecting the marine ecological balance. When they block the mesh of mariculture cages, they can cause large-scale death of fish and shrimp. For a long time, the method to solve the pollution of marine organisms mainly depended on the toxic effect of heavy metal ions, which also causes serious pollution of the marine environment. In recent years, the research and development of nanozyme provides a new solution to prevent and remove marine biological fouling.

**Figure 9:**
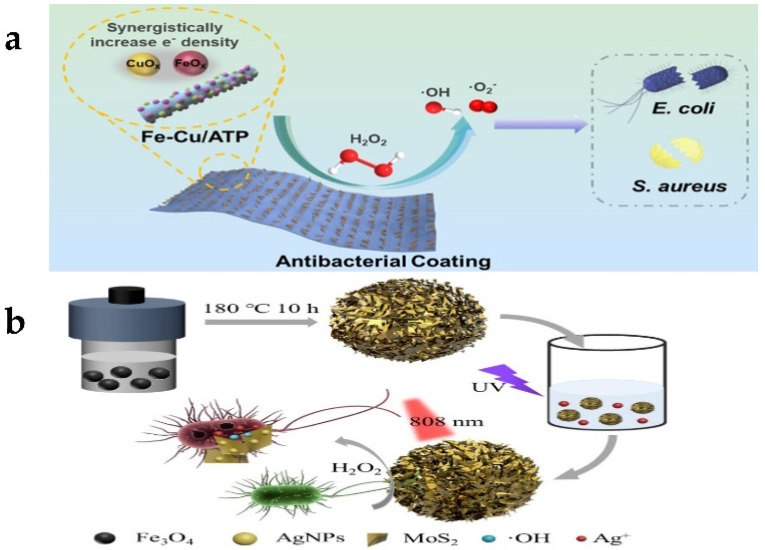
(**a**) Attapulgite doped with Fe and Cu nanooxides as peroxidase nanozymes for antibacterial coatings [189]. (**b**) The schematic preparation of Fe_3_O_4_@MoS_2_-Ag with antibacterial function [190]. Reproduced with permission from (**a**) Ref. [189], Copyright 2022 American Chemical Society; (**b**) Ref. [190], Copyright 2021 Elsevier.

A semiconducting nanozyme consisting of chromium single atoms coordinated on carbon nitride (Cr-SA-CN) that performs bifunctional roles of nonsacrificial H_2_O_2_ photosynthesis and haloperoxidase-mimicking activity for antibiofouling was constructed [186]. The bifunctional Cr-SA-CN nanoplatform promotes the sustainable formation of HOBr under visible light radiation, so it has excellent antibacterial ability. Moreover, the nanozyme can continuously produce H_2_O_2_ from underwater and oxygen under visible light irradiation for enzymatic reaction. Field tests in seawater show that Cr-SA-CN, as an antibacterial additive for environmental protection coatings, can prevent the colonization of marine microorganisms on inert surfaces. In addition, the disinfection efficiency of Cr-SA-CN + Br^−^ against Escherichia coli, Staphylococcus aureus, Pseudomonas aeruginosa, and Vibrio vulnificus was, respectively, 97%, 96%, 92%, and 95%. This study not only proved the ability of monatomic nanozyme to resist biological pollution and sterilization but also provided a strategy for designing more innovative nanozymes with multifunctionality. Nanozyme antifouling agents with the same principle also include photothermal nanozyme with Mo single atom as the active site (Mo SA-N/C), which also has halogen oxide enzyme-like activity [187]. It catalyzes the oxidation of Br^–^ and H_2_O_2_ to produce cytotoxic HOBr. At the same time, the photothermal effect induced by visible light greatly accelerates the reaction process. Attapulgite (ATP) is a kind of natural and available nanomineral that has a special layered chain structure, large specific surface area, strong adsorption capacity, and surface dynamic properties. It provides rapid mass transfer and abundant accessible sites for efficient catalytic reactions [188]. Feng et al. [189] synthesized iron and copper-doped ATP (Fe Cu/ATP) with POD-like activity. The addition of Fe and Cu improves the conversion efficiency of H_2_O_2_, thus showing enhanced POD-like activity. The bactericidal mechanism is to produce reactive oxygen species to attack bacterial populations (Figure 9a). The antibacterial rate of Fe Cu/ATP against Escherichia coli and Staphylococcus aureus is 100% and has a long-term effect. Wei et al. [190] synthesized Fe_3_O_4_@MoS_2_-Ag made great efforts in bacterial adsorption and toxic attack. Topological structure of MoS_2_ nanosheets and S-vacancy enabled Fe_3_O_4_@MoS_2_-Ag to have strong adhesion with bacteria by forming chemical bonds, which shortens the diffusion distance of free radicals and enhances the antibacterial effect (Figure 9b). The nanocomposite has the characteristics of peroxidase simulation and can catalyze H_2_O_2_ to produce living oxygen to attack bacteria. In addition, the released Ag^+^ plays an auxiliary role while attacking the bacterial membrane. Under near-infrared radiation, local hyperthermia and peroxidase simulation can further enhance the sterilization effect. Magnetism also makes it reusable. The method has broad-spectrum antibacterial performance against Gram-negative bacteria, Gram-positive bacteria, drug-resistant bacteria, and fungal bacteria.

### 6.3. Enzyme-like Nanomaterial (Nanozyme)-Based Biofuel Cells

**Figure 10:**
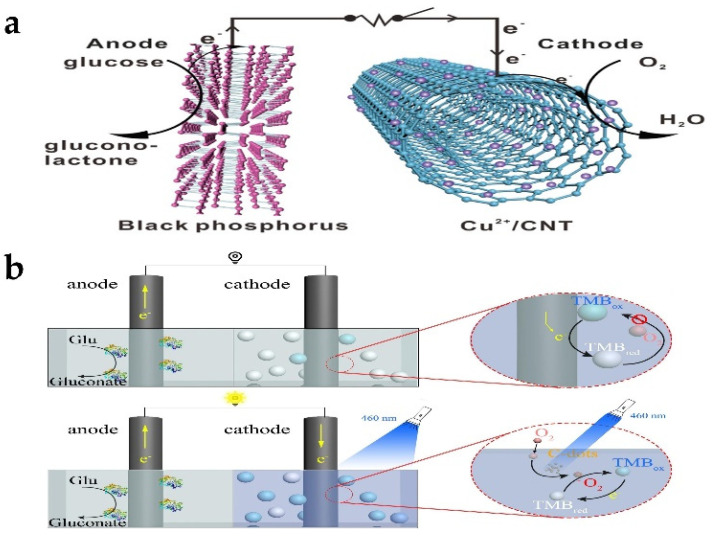
(**a**) Schematic illustration of the as-constructed EBFCs based on BP anode for glucose oxidation and Cu^2+^/CNT cathode for O_2_ reduction [195]. (**b**) Diagram of the photo-switch working mechanism based on C-dots and TMB [197]. Reproduced with permission from (**a**) Ref. [195], Copyright 2020 American Chemical Society; (**b**) Ref. [197], Copyright 2022 Elsevier.

Human beings are facing environmental problems caused by the excessive exploitation of fossil energy. In recent years, countries all over the world have focused on sustainable and environment-friendly new energy. Biofuel cells have become an alternative energy conversion device [191]. Biofuel cells can be divided into three categories: microbial biofuel cells (MBFC), enzyme biofuel cells (EBFC), and enzyme-like nanomaterial (nanozymes)-based biofuel cells (NBFC). MBFC have many advantages in waste treatment and environmental protection [192], but the key disadvantage limiting their wide application and commercialization is that their power output is significantly low, and they are extremely difficult to control the internal electron transfer of microorganisms. Unlike MBFC, EBFC catalyze the oxidation of biofuels to generate electricity with the help of natural enzymes. The biofuels of EBFC are usually sugar relatives, such as glucose, sucrose, fructose, alcohols (including ethanol and methanol), organic acids, and organic salts (such as sulfite). However, glucose-based EBFC have many limitations derived from natural enzymes, such as variability and instability, high production cost, and difficult electron transfer [193,194]. In this case, compared with natural enzymes, nanozymes have become potential catalytic materials for developing glucose biofuel cells due to their inherent characteristics (such as long-term stability, easy synthesis, low cost, and adjustable enzyme mimic activity). Gu et al. [195] found that black phosphorus (BP) showed glucose dehydrogenase (GDH)-like activity and could catalyze the oxidation of glucose without any by-products. BP is a strong alternative candidate for sustainable biofuel cells. Finally, nanozyme-based EBFC consisting of BP anodes for glucose oxidation and Cu^2+^/carbon nanotube (Cu^2+^/CNT) cathodes for reducing O_2_ under natural conditions were successfully constructed (Figure 10a). The power output of BP-based EBFC is higher than that of GDH-based EBFC. BP nanosheets maintained structural integrity before 360 °C, while the protein structure of GDH was destroyed after 250 °C. More important, EBFC based on nanozyme still showed high stability after 30 days of operation. This work provides more possibilities for the application of BP in the field of nanozyme. Compared with metal-based nanozymes, metal oxide nanozymes can be easily synthesized at low cost. Ho et al. [196] proposed interesting NBFC based on metal oxides, in which CoMn_2_O_4_/carbon was used as a GOx-like anode catalyst in biofuel cell systems. NBFC show a power output of 2.372 mW/cm^2^, which is comparable to the commercial platinum/carbon-based biofuel cell system.

In the traditional design of EBFC, once the fuel is added to the anode, the generation of electric energy will start and continue until the fuel is exhausted or the circuit is cut off. This uncontrollable way will lead to energy waste when EBFC are not used. Li et al. [197] proposed a novel optical switch for EBFC by controlling the electron acceptor in the cathode. When the stable TMBred was oxidized by singlet oxygen activated by the C point under light conditions, the medium can accept the electrons generated by the enzyme anode, thus leading to the formation of a path for the external circuit. Without radiation, TMBred cannot be converted into TMB198ox. A limited amount of electron acceptors is rapidly depleted, resulting in almost zero current and power density. Here, the C-point nanozyme was used as a photosensitizer of oxygen, and TMB is added to the cathode chamber as an electron acceptor. Theoretically, without lighting, there should be no current in the external circuit, showing a completely “off” state. The EBFC can be precisely and easily adjusted by the optical switch with light as the input signal (Figure 10b). Therefore, it avoids the damage to the anode enzyme caused by the traditional pH switch. This optical switch can adjust the power output of EBFC according to specific optical signals and promote the “intelligent” application of EBFC.

Enzymes such as nanomaterial (nanozyme)-based biofuel cells mainly use noble metal-based nanozymes, metal oxide-based nanozymes, and electron-receiving laccase that can mimic natural GOx and catalase. NBFC are stable and have a long service life. The high catalytic activity of glucose oxidation can output enough power, which can be synthesized on a large scale and has low production cost. The use of nanozymes in glucose biofuel cell systems has significantly improved the power generation performance. The utilization of NBFC will continue to improve.

## 7. Conclusions and Prospects

To summarize, we reviewed the application of nanozyme in the environmental field from three aspects: environmental monitoring, environmental management, and other environmental applications. The influencing factors of nanozyme catalytic activity were briefly summarized as well. The size, structure, composition combination, doping, and surface modification of nanozyme can adjust the catalytic activity of nanozyme. The specificity of nanozyme is mainly improved by biological recognition molecules (biological enzyme, MIP, DNA, antibody, aptamer) and the simulation of the active center and binding site of the biological enzyme. In addition to its own intrinsic enzyme activity, it can also affect the catalytic efficiency through pH, temperature, light stimulation, and so on. In terms of environmental monitoring and treatment, the detection and degradation of heavy metal ions, phenolic compounds, dyes, plasticizers, pesticides, and antibiotics all involve nanozymes. Moreover, nanozymes can be used as antibacterial and antifouling agents and biofuel cells to indirectly protect the environment. Great development and applications of nanozyme have been promoted in the field of environmental science. 

Nanozyme has been developed for more than 20 years. Nanomaterials with new enzyme activities have been continuously explored. The design strategy of nanozymes has been constantly improved. However, nanozyme still has some limitations regarding the direction of development in the future.

- At present, the types of nanozymes are still too few, and they are mainly concentrated in the oxidoreductase family and hydrolase family. Compared with the six categories of natural enzymes, there is still an urgent need to unlock more simulated enzymes with different catalytic activities to expand the scope of application.
- Nanozymes are a succedaneum for natural enzymes, but the catalytic activity of most nanozymes is far inferior to natural enzymes. Hence, strategies need to be continuously explored to improve their catalytic activity.
- Nanozymes can show good performance in the laboratory. Nevertheless, they are still disadvantaged because they cannot be used on a large scale for the actual pollutant treatment industry, such as catalytic devices requiring high-precision technology, short service life, and higher cost than traditional environmental treatment methods.
- Although some nanozymes that break through the restriction of pH have appeared, most nanozymes are still limited by pH with narrow range. Technological breakthroughs are still needed in this regard so that the catalytic activity of most nanozymes is no longer limited by pH.
- Nanozymes are intrinsically toxic. It is vital to design low-toxicity nanozymes by adjusting their physical and chemical properties such as size, shape, surface properties, surface charge, and chemical composition to avoid secondary contamination.
- In recent years, nanozymes with multienzyme activity have been continuously developed, which can be used for multifunctional applications. However, at the same time, facing the challenge that the selectivity of nanozymes with multienzyme activity is lower than that of single-enzyme live nanozymes, will challenge researchers to balance the relationship between "multifunction" and “high selectivity” as well as achieve a win–win situation.
- Recently, the specificity of nanozyme is much lower than that of natural enzyme. The design of nanozyme should be committed to better a bionic biological enzyme’s active center and binding site, and the recognition element should be stably and effectively connected to the nanozyme. In addition, it is critical to explore the mechanism and law of interactions between nanozyme and a recognition element. Meanwhile, it is still necessary to improve the performance of nanozyme sensors by combining the research results of specific recognition in other fields and sensing technologies.
- Nanozyme detection mostly relies on colorimetric sensing, but colorimetric sensing has the problems of large interference and low sensitivity. In addition to electrochemistry, photoelectrochemistry, and surface-enhanced Raman scattering (SERS), adding more detection modes can have unexpected effects on environmental monitoring.

## Figures and Tables

**Table 2:** Summary of the application of nanozymes in environmental management.

Category	Pollutant	Activity	Nanozyme	Removal Efficiency	Ref.
Dyes	RhB	POD	Sulfur-doped graphdiyne	>98%	[159]
	Methyl orange	POD	CNZ	93%	[160]
	Rhodamine B	OXD	FeBi-NC SAzyme	99%	[161]
	Methylene Blue	POD	ZnNi-MOF/GO NCs	95%	[162]
	Methylene Blue	POD	Cu^2+^-HCNSs-COOH	80.7%	[163]
	Methylene Blue	POD OXD	PdNPs/PCNF	99.64%	[151]
	Amido Black	Laccase	Cu/H_3_BTC MOF	60%	[164]
	Malachite green	Laccase	Fe_3_O_4_@C-Cu^2+^	99%	[165]
	Organic dyes	POD	Fe_3_O_4_@Gel	99%	[166]
Antibiotics	Tetracycline	POD	Sulfur-doped graphdiyne	>69%	[159]
Toxic ions	Cr^6+^/As^3+^	CAT	NanoMn_3_O_4_	>98%	[167]
	Hg^2+^/Cl^−^	POD	AgRu@β-CD-co-GO	94.9% 93.8%	[168]
	H_2_O_2_	CAT POD	DMNS@AuPtCo	>95%	[169]
Phenolic	Hydroquinone	Laccase	Aminopropyl-functionalized copper containing phyllosilicate (ACP)	100%	[170]
	Phenol	POD	MNP@CTS	>95%	[171]
	Phenol	CAT POD	DMNS@AuPtCo	90%	[169]
	2,4-DP	Laccase	Fe1@CN-20	65%	[172]
	2,4-DP	Laccase	AMP-Cu	65%	[124]
	2,4-DP	Laccase	CH-Cu	82%	[173]
	2,4-DP	Laccase	Cu-Cys@COF-OMe	>75%	[174]
	2,4-DP	Laccase	CA-Cu NPs	90%	[175]
	DEHP phthalic acid esters	Hydrolase	Zn-heptapeptide bionanozymes	86.80%	[176]
	Microplastics	POD	Fe_3_O_4_NPs	100%	[177]
Pathogens	*Escherichia coli*	Phospholipase	PAA-Cnp	>80%	[178]
	*Escherichia coli*	POD	Au-Pt dumbbell NPs	95%	[157]
	*Escherichia coli*	OXD	w-SiO_2_/CuO	90%	[179]
	*Gram-negative bacteria*	POD	SA-Pt/g-C_3_N_4_-K	>99.99%	[180]
OPs	Simazine	POD	Fe_3_O_4_/DG	99%	[181]
	Atrazine	POD	Fe_3_O_4_-TiO_2_/rGO	98%	[141]
	Cinosulfuron	POD	CP@CA	96.25%	[182]
